# Simulating phenolic acid input enhances rice phosphorus uptake by increasing the relative abundance of *Diversispora*

**DOI:** 10.3389/fmicb.2026.1810465

**Published:** 2026-04-29

**Authors:** Yao Cheng, Guiwei Wang, Yuanyuan Yao, Lu Liu, Zekun Wang, Yuying Yan, Yixuan Liang, Wanting Qi, Yuechao Yang

**Affiliations:** National Engineering Research Center for Efficient Utilization of Soil and Fertilizer Resources, National Engineering & Technology Research Center for Slow and Controlled Release Fertilizers, College of Resources and Environment, Shandong Agricultural University, Taian, Shandong, China

**Keywords:** cumalic acid, *Diversispora*, fungi, phosphorus, rice

## Abstract

**Introduction:**

Phenolic acids are allelopathic substances that mediate plant-fungal interactions. However, the regulatory effects of phenolic acids on soil microbiota and crop phosphorus (P) uptake remain unclear. Thus, this study aimed to investigate the effects of continuous addition of cumalic acid (CA) on soil microbial communities and P uptake of rice.

**Methods:**

We continuously added different concentrations of CA to the rhizosphere during the rice growth to investigate its effects on soil fungal communities and P uptake of rice.

**Results:**

The addition of 5 and 10 μmol kg^−1^ CA significantly increased P uptake in rice by 51.4% and 63.8%, respectively. In addition, the 10 μmol kg^−1^ CA treatment significantly increased soil Olsen P content. Exogenous addition of CA altered the diversity and composition of soil microbial communities. The application of 5 and 10 μmol kg^−1^ CA significantly elevated the relative abundance of Diversisporales from 46.3% to 68.7% and 67.1%, respectively; while suppressing Eurotiales from 15.8% to 2.6% and 5.7%, respectively. At the genus level, the treatment enhanced *Diversispora* (from 46.3% to 68.7% and 67.0%) and reduced *Penicillium* (from 9.0% to 1.2% and 2.2%). Notably, the relative abundance of *Diversispora* was significantly and positively correlated with P uptake of rice, whereas *Penicillium* was negatively correlated with P uptake.

**Conclusion:**

CA stimulated the P uptake of rice by shaping keystone fungal taxa. Our results suggest that the indirect microbial-mediated mechanism dominated P uptake improvement of rice. This study provides critical insights into the potential application of allelopathic substances in agricultural production.

## 1 Introduction

Soil microbes play an indispensable role in cycling soil nutrients, mitigating pathogen and pest damage, and promoting plant nutrient uptake (Backer et al., 2018). In the rhizosphere, the interactions between root exudates and the soil environment form a complex root microbiome (Trivedi et al., 2020). Plant-associated bacteria and fungi participate in various metabolic activities affecting soil phosphorus (P) turnover (Zheng et al., 2025; Sun N. et al., 2025). In particular, the proportion of microbes that are significantly influenced by root exudates often plays a key role in maintaining plant nutrition and health (Wu et al., 2025; Wang et al., 2022). Therefore, an in-depth investigation of rhizosphere microbial communities and their functions is crucial for elucidating the mechanisms underlying plant-soil-microbe interactions.

Generally, plant root exudates contain sugars, organic acids, flavonoids, phenolics, and various other compounds (Bulgarelli et al., 2013). They act as chemical signals and carbon sources within the rhizosphere microbiome, significantly influencing the composition, diversity, and function of soil microbes (Hu et al., 2018). For example, certain low-molecular-weight organic acids, such as oxalic and citric acids, can stimulate P-solubilising bacteria, enhancing the dissolution of inorganic P and the mineralization of organic P in the soil (Wang et al., 2023; Koester et al., 2021). Moreover, succinic acid significantly increases maize P uptake by enriching amplicon sequence variants (ASVs) associated with Actinomycetes and Proteobacteria (Wang et al., 2025a). Similarly, adding luteolin, a common maize exudate, increases the relative abundance of Gaiellales, which is positively correlated with shoot P content and soil phosphatase activity, suggesting its potential role in P mobilization (Wang et al., 2025b). Hence, harnessing root exudates to regulate the phosphate-solubilising function of soil microbes could be an effective approach to enhance crop P uptake. This approach also provides valuable insights into leveraging the biological potential of soil microbes to improve crop productivity in future agricultural practices (Mendes et al., 2013).

Phenolic acids, also known as allelochemicals, primarily exert their ecological effects by inhibiting weeds and pathogens (Xie et al., 2023). With advancing research, phenolic compounds also function as signaling molecules that regulate microbial metabolic activities, influence the composition and function of soil microbial communities, and modulate nutrient transformation and availability by altering the chemical properties of the rhizosphere (Zhang et al., 2019; Berendsen et al., 2018). For instance, *p*-hydroxybenzoic acid can recruit and stimulate phenolic acid-degrading specialists, such as *Paraburkholderia*, whose metabolism triggers a priming effect that significantly accelerates the mineralisation of native soil organic carbon, thereby exerting a profound impact on the soil carbon turnover (Wilhelm et al., 2021). Mycorrhizal colonization promotes soybean nodulation and nitrogen fixation by upregulating the expression of the *GmPAL2.4* gene, which consequently drives the root exudation of key phenolic acids such as benzoic acid and *p*-hydroxybenzoic acid, thereby enhancing rhizobial growth, chemotaxis, and biofilm formation (Li et al., 2025). As allelochemicals, phenolic acids can modulate soil fungal communities to enhance plant disease resistance. For example, healthy American ginseng plants secrete high levels of phenolic acids, particularly the key compound *p*-coumaric acid (Zhang et al., 2023). This secretion specifically enriches beneficial biocontrol fungi, such as *Trichoderma* spp., while suppressing the proliferation of pathogenic *Fusarium* spp., thereby steering the microbial community toward a composition that favors plant health (Zhang et al., 2023). These studies highlight the importance of phenolic acids in plant–soil–microbe interactions. However, the mechanisms by which phenolic acids influence rice P uptake and utilization through the regulation of microbial activity and soil P bioavailability remain unclear.

Under drought stress, colonization by the root endophytic fungus *Serendipita indica* increases cumalic acid (CA) content in *Pinus taeda* seedlings by more than threefold (Wu et al., 2024). However, it remains unclear whether this accumulation directly influences plant nutrient metabolism. In the present study, we aimed to investigate the effects of continuous CA application on P uptake in rice. We explored whether applying different concentrations of CA to the rhizosphere could enhance soil organic P utilization by altering fungal community structure. We hypothesized that high concentrations of CA application would: (1) increase rice P uptake, (2) increase available P content in the rhizosphere soil, and (3) alter the composition and diversity of fungal communities in the rice rhizosphere. This study provides new scientific insights into the role of phenolic acids in plant-soil-microbe interactions and offers theoretical support for optimizing P nutrition management strategies in rice cultivation.

## 2 Materials and methods

### 2.1 Experimental rice and soil

The rice variety tested was “Xudao3.” Seeds were disinfected with a 4% sodium hypochlorite solution and rinsed with sterile deionised water. They were then placed in a dark environment at 25 °C and soaked in sterile deionised water for 48 h before sowing.

Calcareous soil (classified according to the USDA soil taxonomy) was collected from Tai'an (117°05′E, 36°12′N). After air-drying and sieving through a 2 mm mesh. The basic physicochemical properties of the soil were as follows: pH of 7.85 (measured at a 5:1 water/soil ratio), organic matter content of 7.03 g kg^−1^, total N content of 0.45 g kg^−1^, mineral nitrogen (NO3− and NH4+) content of 30.1 mg kg^−1^, Olsen-extractable P content of 6.0 mg kg^−1^ (extracted with 0.5 M NaHCO3), and CH3COONH_4_-extractable K content of 109 mg kg^−1^.

### 2.2 Experimental design

The following nutrients were added to the air-dried soil to supplement the mineral content per kilogram of soil: 200 mg N (as urea), 20 mg P (as KH_2_PO_4_), and 200 mg K (as K_2_SO_4_). Pots with an upper diameter of 21.4 cm, a lower diameter of 19.1 cm, and a height of 21 cm were used. Each pot was filled with 5 kg of air-dried soil. One end of the silicone tubing (2 mm inner diameter, 15 cm length) was inserted below halfway point of the soil in each pot for the subsequent addition of CA solutions. Three silicone tubes were evenly distributed in each pot, and 1,500 mL of sterile water was added to thoroughly moisten the soil. After 7 days, the rice seedlings were thinned to retain four plants per pot. Flooded conditions were maintained throughout the rice growth period under natural light conditions from 27 June to 16 August 2024, spanning 7 weeks, with temperatures ranging from 16 °C to 37 °C. The experiments were conducted at Shandong Agricultural University, Tai'an City, Shandong Province.

CA solutions at concentrations of 5 and 10 μmol kg^−1^ soil for rhizosphere application were prepared using sterile water. Sterilization was achieved using syringes fitted with 0.2 μm filter membranes during solution addition. During the 3-7 weeks following sowing, CA solutions were applied to the soil every 3 days (total amounts are 0, 225, and 450 μmol respectively), with sterile water applied to the control plots concurrently.

The experiment employed a completely randomized block design with three treatments: control group without CA addition; low-concentration group with 5 μmol kg^−1^ soil; and high-concentration group with 10 μmol kg^−1^ soil. Each treatment was replicated four times.

### 2.3 Sample collection, P uptake, available P determination, and DNA extraction

After 5 weeks of treatment, the aboveground parts of the rice plants and the rhizosphere soil (0–2 mm) were collected. The aboveground parts of each rice plant were cut and placed in a 105 °C oven for 30 min and then dried at 75 °C to constant weight. The dried samples were ground and sieved, and P uptake was determined using the method described by Thomas et al. (1967). Excess root soil was manually shaken off, leaving approximately 2 mm of the rhizosphere soil adhered to the roots. The rhizosphere soil samples were collected, air-dried, and sieved through a 2 mm mesh. Available P in the rhizosphere soil was extracted using 0.5 M NaHCO_3_ following the method described by Olsen (1954). Available P content was determined using the molybdenum blue colorimetric method with UV-visible spectrophotometry. Another portion of the rhizosphere soil sample was stored at −80 °C for subsequent DNA analysis. DNA was extracted from 0.50 g of soil using the MagBeads FastDNA Kit for Soil (MP Biomedicals, Santa Ana, CA, USA), following the manufacturer's instructions. The extracted DNA was stored at −20 °C for real-time qPCR analysis and sequencing preparation.

### 2.4 ITS rRNA gene amplicon sequencing

The quantity and quality of the extracted DNA were measured using a NanoDrop NC2000 spectrophotometer (Thermo Fisher Scientific, Waltham, MA, USA) and agarose gel electrophoresis, respectively. The PCR amplification of the fungal ITS rRNA gene V1 region was performed using the forward primer ITS1F (5′- CTTGGTCATTTAGAGGAAGTAA-3′) and the reverse primer ITS2 (5′- GCTGCGTTCTTCATCGATGC-3′). Upon assembling the requisite components for the PCR reaction, the template DNA was first subjected to a pre-denaturation step at 98 °C for 5 min using a thermal cycler to ensure complete denaturation. The reaction then proceeded to the amplification phase, consisting of 30 cycles. Each cycle included denaturation at 98 °C for 30 s, annealing at 55 °C for 45 s to facilitate primer–template hybridization, and extension at 72 °C for 45 s to enable DNA synthesis. Following the cycling phase, a final extension was carried out at 72 °C for 5 min to ensure complete elongation of amplification products, after which samples were held at 12 °C for storage.

The amplification products were analyzed using 2% agarose gel electrophoresis. Target DNA bands were excised and purified using the Axygen gel extraction kit. After individual quantification, amplicons were pooled in equal amounts and subjected to paired-end 2 × 250 bp sequencing on the Illumina NovaSeq platform using the NovaSeq 6,000 SP Reagent Kit. Bioinformatics analysis of the microbiome was performed using QIIME2 (Bolyen et al., 2018). In brief, raw sequence data were demultiplexed using the demux plugin, followed by primer trimming using the cutadapt plugin (Martin, 2011). The sequences were then quality filtered, denoised, merged, and subjected to chimera removal using the DADA2 plugin (Callahan et al., 2016). At last, flattened ASV tables were generated to record the abundance of each ASV in each sample and the taxonomy of these ASVs.

### 2.5 Statistical analyses

All datasets were assessed for normality (Kolmogorov–Smirnov test) and homogeneity of variances (Levene's test) using IBM SPSS Statistics 27 before performing parametric analyses. We then applied one-way ANOVA to evaluate the effects of treatments on shoot P content, Olsen P, Simpson index, and Shannon index. For variables with significant ANOVA results, Duncan's test was used for *post-hoc* comparisons. Significant differences were determined at *P* ≤ 0.05.

Fungal ASVs that were common or unique to the different treatments were identified and visualized using Venn diagrams, which were generated using the R package “VennDiagram” based on common and unique data (Zaura et al., 2009). Spearman correlation analyses were employed to evaluate potential linkages between the top nine rhizosphere fungal genera and rice shoot P uptake.

The samples, which were stratified into three concentration groups (Control, 5 μmol kg^−1^ CA, and 10 μmol kg^−1^ CA), constituted the entire dataset for subsequent analysis. A training set was established from this dataset by randomly selecting 75% of the samples from each group. A random forest model was built using this training set to classify the concentration groups. The importance of each ASV for accurate group prediction was quantified by the increase in the model's mean squared error when its abundance data were permuted (Edwards et al., 2018). Model refinement was achieved by 10-fold cross-validation integrated with recursive feature elimination (using the rfcv() function), which assessed performance as ASVs were sequentially removed. The correlation between the relative abundance of the top 10 ASVs and shoot P content was evaluated using Spearman's rank correlation method. All statistical analyses were performed using R Studio (R version 4.0.5, http://www.r-project.org) unless otherwise indicated.

## 3 Results

### 3.1 Effects of CA on rice shoot P and rhizosphere soil Olsen P

**Figure 1:**
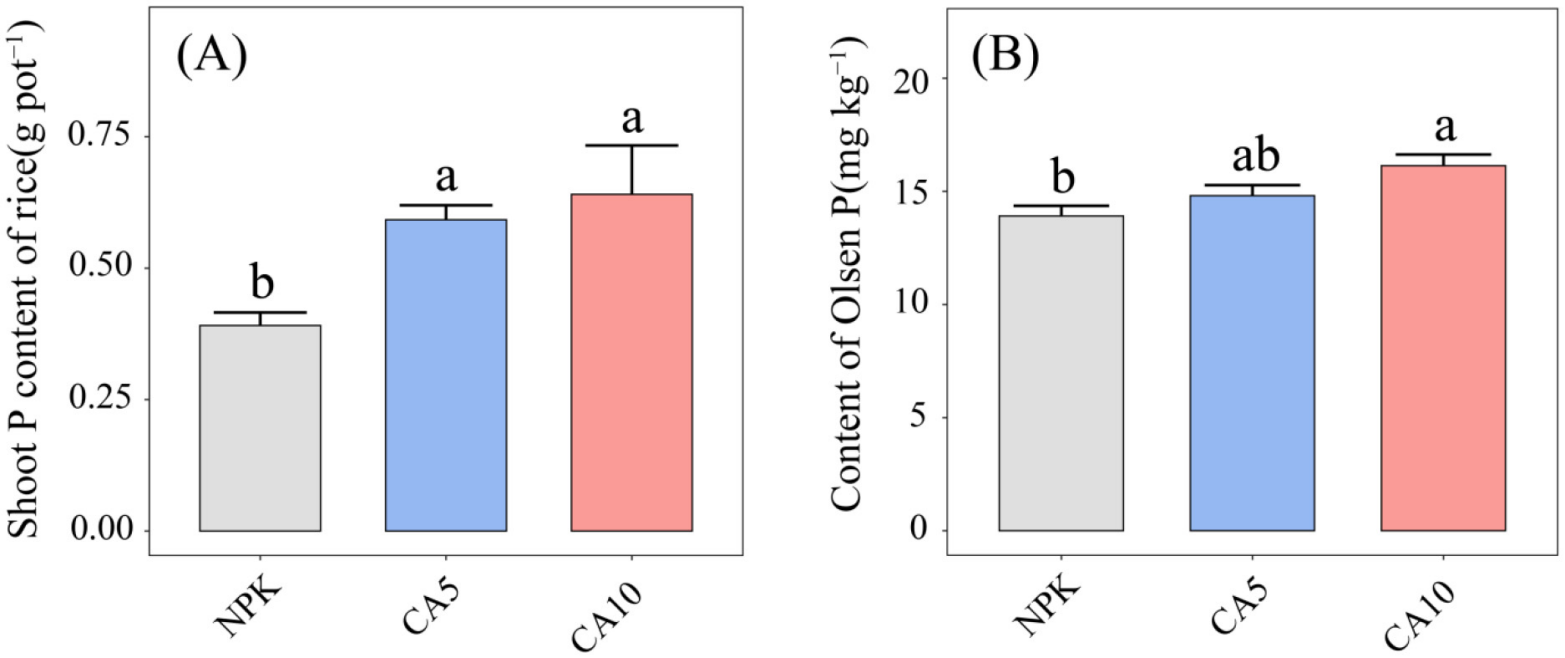
**(A)** Shoot P content of rice, **(B)** Olsen P of rhizosphere soil at different treatments. Different lowercase letters indicate significant differences among different treatments (Duncan's test at *P* ≤ 0.05). Data are means + SE (*n* = 4). NPK, control; CA5, cumalic acid at 5 μmol kg^−1^; CA10, cumalic acid at 10 μmol kg^−1^.

CA addition significantly influenced both shoot P content in rice and Olsen P content in the rhizosphere soil, with the 10 μmol kg^−1^ CA treatment yielding the most pronounced effects (Figure 1). Compared to the NPK treatment, the 5 and 10 μmol kg^−1^ CA treatments significantly increased shoot P content by 51.4% and 63.8%, respectively (Figure 1A). In addition, the 10 μmol kg^−1^ CA treatment significantly increased soil Olsen P content (Figure 1B).

### 3.2 Alpha diversity index and beta diversity index

**Figure 2:**
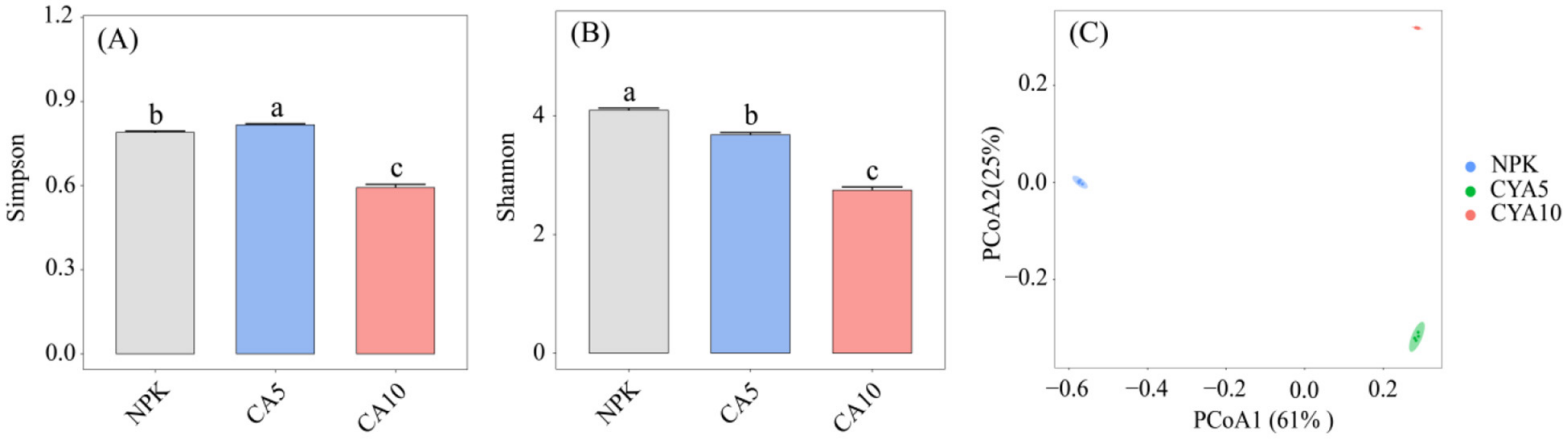
**(A)** Simpson index of soil fungi under different treatments, **(B)** Shannon index of soil fungi under different treatments, **(C)** principal coordinate analysis. Data are means + SE (*n* = 4). NPK, control; CA5, cumalic acid at 5 μmol kg^−1^; CA10, cumalic acid at 10 μmol kg^−1^.

The addition of CA significantly affected the alpha diversity of soil fungi (Figures 2A, B). Compared to the NPK treatment, the 5 μmol kg^−1^ CA treatment significantly increased the Simpson index but decreased the Shannon index of soil fungi. In contrast, the 10 μmol kg^−1^ CA treatment significantly reduced both the Simpson and Shannon indices. Moreover, principal coordinate analysis showed that the addition of different concentrations of CA notably altered the soil fungal community (Figure 2C).

### 3.3 Fungal community composition

**Figure 3:**
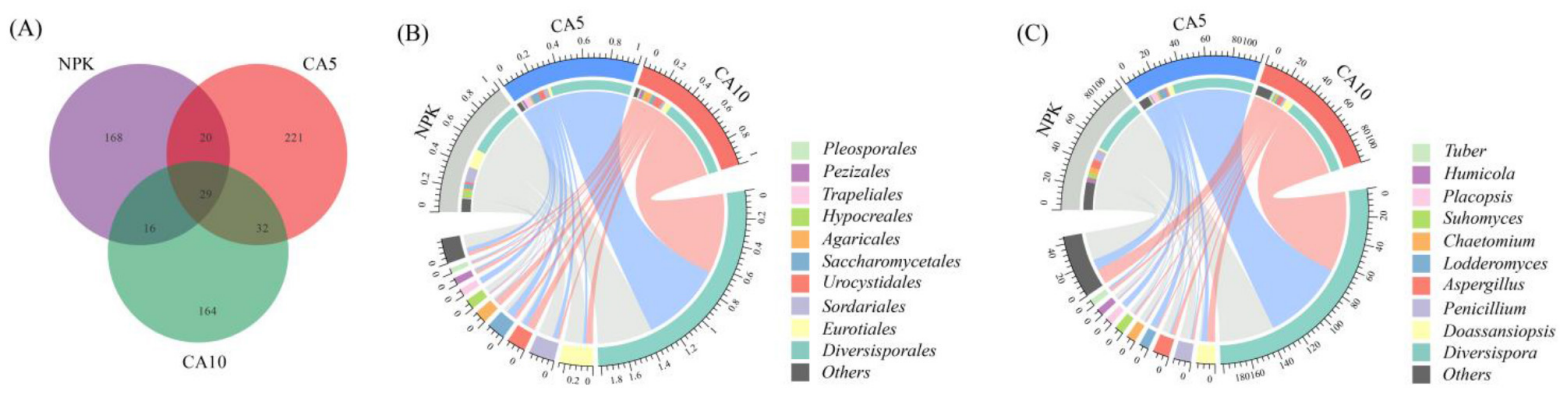
Venn diagram **(A)** and top 10 relative abundance at the order and genus levels of soil fungi order: **B**; genus: **(C)**. Permutational multivariate analysis of variance was used to test the difference in fungal communities among different treatments. The number in each section of the Venn diagram corresponds to the ASV count.

The Venn diagrams of fungi at the ASV level (Figure 3A) showed that the number of shared fungal ASVs across all treatments was 29, and the number of unique fungal ASVs was largest in the 5 μmol kg^−1^ CA treatment. The NPK treatment shared 49 and 45 of the same fungal ASVs with the 5 and 10 μmol kg^−1^ CA treatments, respectively.

The relative abundances of fungal communities at the order and genus levels across the different treatments are depicted in the chord diagrams in Figures 3B, 3C, respectively. As shown in Figure 3B, Diversisporales was the dominant order, followed by Eurotiales, Sordariales, Urocystidales, Saccharomycetales, Agaricales, Hypocreales, Trapeliales, Pezizales, and Pleosporales. Notably, the 5 μmol kg^−1^ CA treatment significantly enhanced the relative abundances of several orders compared with the NPK treatment: Diversisporales (from 46.3 to 68.7%), Urocystidales (from 1.8 to 4.7%), Saccharomycetales (from 3.8 to 6.6%), Trapeliales (from 0.0025 to 5.3%), and Pezizales (from 0.375 to 1.8%). In contrast, the same CA5 treatment significantly reduced the relative abundances of Eurotiales (from 15.8 to 2.6%), Sordariales (from 12.5 to 4.0%), Agaricales (from 1.7 to 0.9%), and Hypocreales (from 5.6 to 0.6%).

When the CA concentration was increased to 10 μmol kg^−1^, a distinct shift in fungal order abundance was observed. This treatment significantly increased the relative abundances of Diversisporales (46.3 to 67.1%), Urocystidales (1.8 to 7.0%), Agaricales (1.7 to 6.2%), Pezizales (0.375 to 3.0%), and Pleosporales (1.1 to 1.5%). Conversely, it significantly decreased the relative abundances of Eurotiales (15.8 to 5.7%), Sordariales (12.5 to 2.2%), Saccharomycetales (3.8 to 3.1%), and Hypocreales (5.6 to 0.9%).

At the genus level, *Diversispora* was the most abundant taxon, followed by *Doassansiopsis, Penicillium, Aspergillus, Lodderomyces, Chaetomium, Suhomyces, Placopsis, Humicola*, and *Tuber* (Figure 3C). Compared with the NPK treatment, the 5 μmol kg^−1^ CA treatment significantly increased the relative abundances of several genera: *Diversispora* (46.3 to 68.7%), *Doassansiopsis* (1.8 to 4.7%), *Lodderomyces* (0.0025 to 5.6%), *Placopsis* (0.0025 to 5.3%), and *Tuber* (0.1 to 1.7%). By contrast, the same treatment significantly reduced the relative abundances of *Penicillium* (9.0 to 1.2%), *Aspergillus* (6.8 to 1.4%), *Chaetomium* (5.0 to 0.8%), *Suhomyces* (3.8 to 1.0%), and *Humicola* (3.7% to 0.8%). Compared with the NPK treatment, the 10 μmol kg^−1^ CA treatment significantly increased the relative abundances of *Diversispora* (46.3 to 67.0%), *Doassansiopsis* (1.8 to 7.0%), *Lodderomyces* (0.0025 to 1.4%), and *Tuber* (0.1 to 2.7%). Conversely, the same treatment significantly reduced the relative abundances of *Penicillium* (9.0 to 2.2%), *Aspergillus* (6.8 to 3.6%), *Chaetomium* (5.0 to 0.9%), *Suhomyces* (3.8 to 1.7%), and *Humicola* (3.7 to 0.5%).

### 3.4 Correlation of the top nine genera with rice shoot P

**Figure 4:**
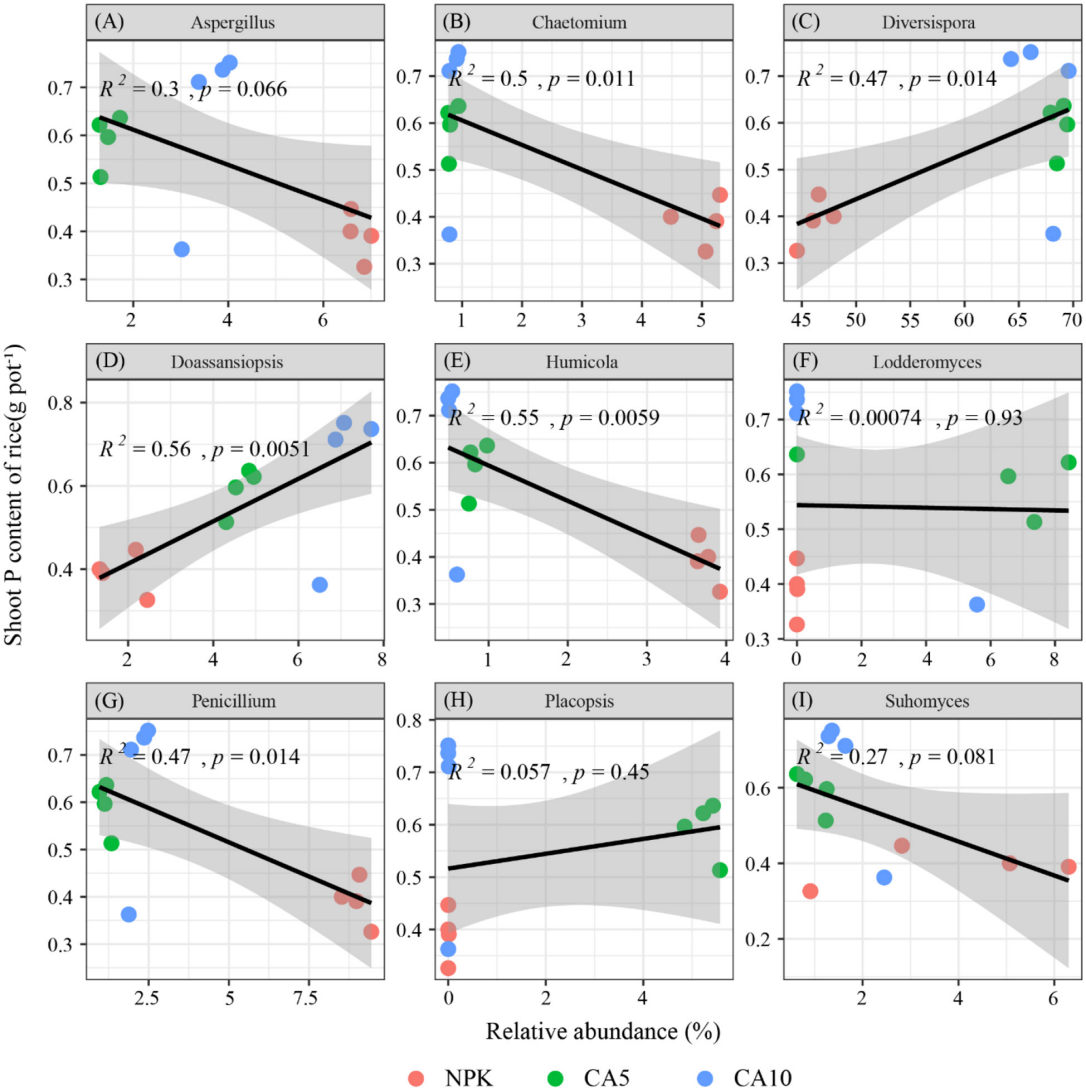
Spearman correlation between the relative abundance of the top nine fungal genera and rice shoot P content under different CA addition concentrations.

At the genus level, the relative abundances of five fungal taxa demonstrated a significant linear correlation (*P* ≤ 0.05) with rice shoot P content. Analysis of the nine most abundant genera revealed distinct correlation patterns (Figure 4). Specifically, *Aspergillus* (Figure 4A) showed no significant association with P content, whereas *Chaetomium* (Figure 4B) exhibited a significant negative correlation. In contrast, two genera displayed positive relationships with rice shoot P content: *Diversispora* (Figure 4C) and *Doassansiopsis* (Figure 4D), both of which were both significantly positively correlated. Returning to negative correlations, *Humicola* (Figure 4E) was also significantly negatively correlated. Conversely, *Lodderomyces* (Figure 4F) showed no significant link, a pattern similarly observed for *Penicillium* (Figure 4G), which was significantly negatively correlated. Finally, the remaining genera, *Placopsis* (Figure 4H) and *Suhomyces* (Figure 4I), consistently showed no significant association with shoot P content.

### 3.5 Important ASVs

**Figure 5:**
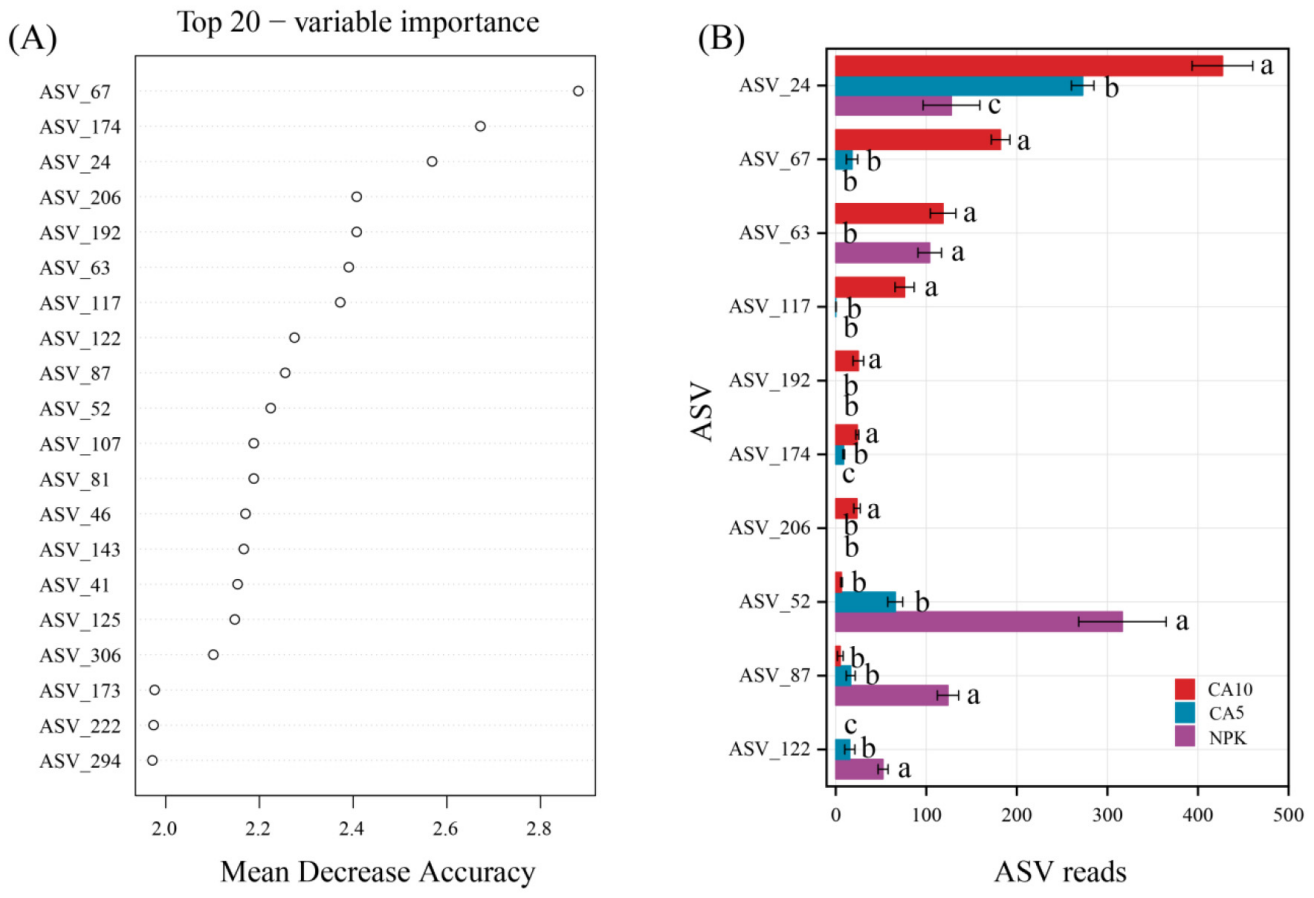
**(A)** Top 20 most important ASVs. **(B)** Ten biomarker ASVs identified by the random forest model. Mean decrease accuracy means accuracy of random forest prediction. Data are means + SE (*n* = 4). NPK, control; CA5, cumalic acid at 5 μmol kg^−1^; CA10, cumalic acid at 10 μmol kg^−1^.

The top 20 most important ASVs were identified using the random forest model (Figure 5A), as shown in Supplementary Table 1. Compared to the NPK treatment, the 5 μmol kg^−1^ CA treatment increased the relative abundances of ASV_24, ASV_67, ASV_117, and ASV_174, and decreased those of ASV_63, ASV_52, ASV_87, and ASV_122. Similarly, the 10 μmol kg^−1^ CA treatment increased the relative abundances of ASV_24, ASV_67, ASV_63, ASV_117, ASV_192, ASV_174, and ASV_206, and decreased those of ASV_52, ASV_87, and ASV_122 (Figure 5B).

### 3.6 Correlations of the top 10 important ASVs with rice shoot P

**Figure 6:**
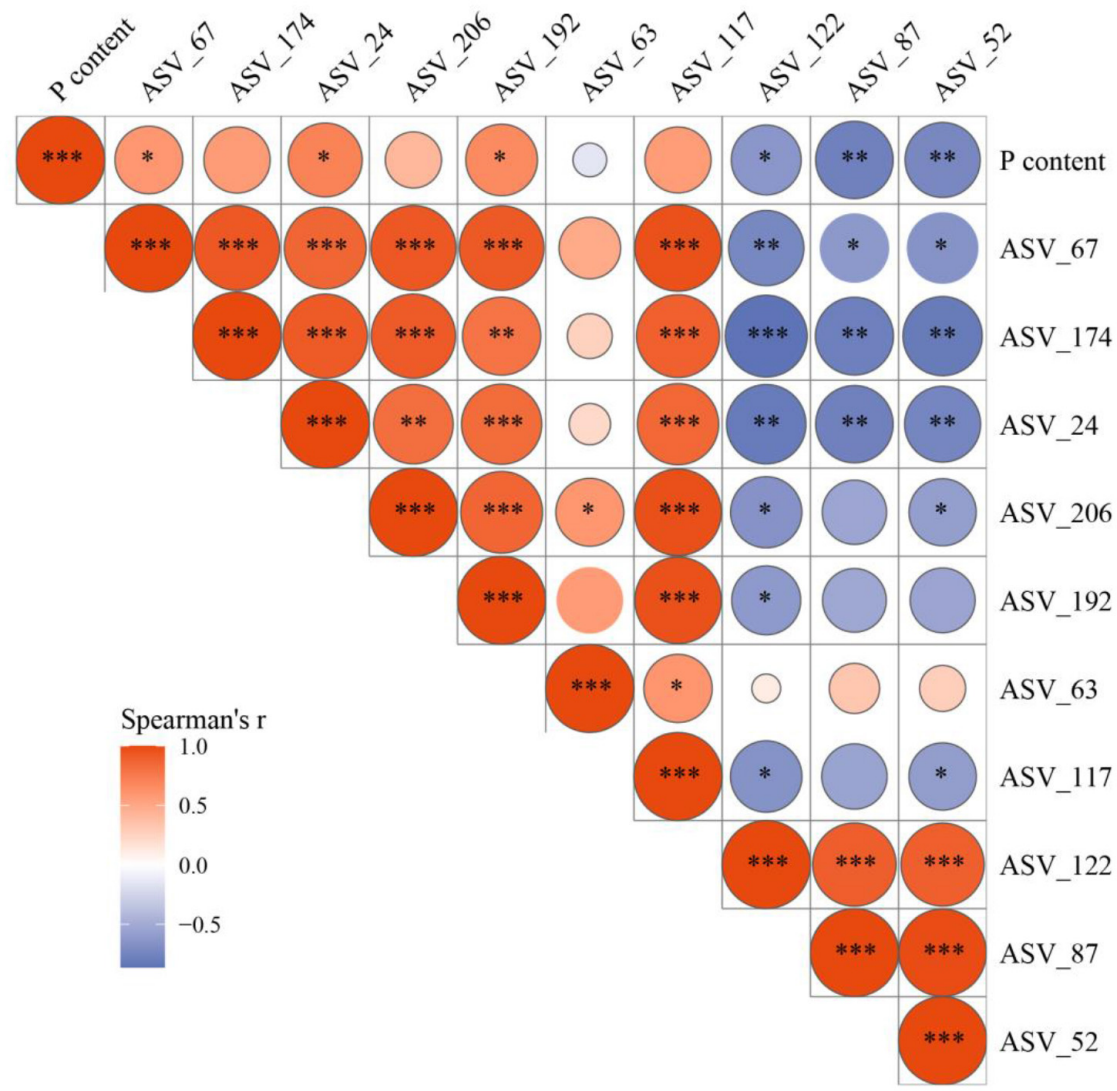
Spearman correlation among the relative abundance of identified the top 10 most important ASVs and shoot P content in rice. The area of the bubble represents the absolute value of the correlation matrix, and the color of the bubble represents the Spearman correlation coefficient. *P ≤ 0.05; **P ≤ 0.01; ***P ≤ 0.001.

Spearman's correlation analysis was used to establish the relationships between the shoot P content in rice and the relative abundances of the top 10 most important ASVs (ASV_67, ASV_174, ASV_24, ASV_206, ASV_192, ASV_63, ASV_117, ASV_122, ASV_87, and ASV_52). These key ASVs were previously identified by the random forest model, thereby representing associations with shoot P content at different levels (Figure 6). The relative abundances of ASV_67, ASV_24, and ASV_192 showed a significant positive correlation with rice shoot P content, whereas those of ASV_122, ASV_87, and ASV_52 exhibited a significant negative correlation with rice shoot P content.

## 4 Discussion

### 4.1 CA promotes P uptake in rice

The interaction between plants and microorganisms largely relies on the rhizosphere chemical dialogue, in which root exudates play a central role (Lebeis et al., 2015; Hu et al., 2018). In this work, exogenous application of CA, a typical phenolic acid signaling molecule, significantly enhanced P uptake in rice seedlings (Figure 1A). Notably, the indirect microbial-mediated mechanism dominated this P uptake improvement, whereas the direct P-mobilizing capacity of CA was relatively restricted due to its structural characteristics. Directly, CA contains only one carboxyl functional group, which limits its ability to chelate insoluble P-metal complexes or acidify the rhizosphere soil for P mobilization. Although CA could slightly increase soil available P content via weak carboxyl-mediated chelation or mild acidification (Figure 1B), this direct effect is far less efficient than that of low-molecular-weight organic acids with multiple carboxyl groups (e.g., citric acid, benzoic acid) (Zheng et al., 2025; Sokolova, 2020). Thus, the direct P-solubilizing role of CA is considered a secondary and auxiliary mechanism in this process. Indirectly and critically, as the dominant phenolic root exudate, CA functions as a key signaling cue to orchestrate rhizosphere fungal communities that are tightly linked to enhanced P utilization efficiency in rice (Li et al., 2025). Exogenous CA supplementation dramatically reshaped the rhizosphere fungal community composition, markedly elevated fungal alpha diversity (Figure 2), and specifically enriched beneficial fungal taxa closely associated with inorganic P solubilization, organic P mineralization, and mycorrhizal symbiosis. These CA-modulated beneficial fungi form a highly efficient root-mycorrhizal-microbial synergistic system that facilitates P mobilization, translocation, and uptake in rice. Collectively, the indirect regulation of rhizosphere beneficial fungal communities represents the core and decisive mechanism underlying CA-improved rice P uptake, far outweighing the limited direct P-mobilizing effect of CA.

### 4.2 Effects of CA on fungal communities and functions

The addition of CA significantly enriched key arbuscular mycorrhizal fungi (AM fungi) groups that are positively correlated with P uptake, such as *Diversispora* (Figures 3 extbfand 4) (Weber et al., 2025). The arbuscular hyphal network of AM fungi, as a ‘highway' in the soil, can not only greatly expand the uptake range of roots but also serve as a channel for the transmission of signals/nutrients and the movement of phosphate-solubilising bacteria, directly enhancing the ability of rice to obtain soil P (He et al., 2024; Liu et al., 2025). Simultaneously, the addition of CA significantly reduced the relative abundances of multiple potentially pathogenic bacterial genera, including *Penicillium* and *Humicola* (Figure 3), which were significantly negatively correlated with P uptake (Figure 4). The decrease in their abundances directly reduces the competitive pressure of fungi for P resources and inhibits their potential pathogenicity, creating a more favorable rhizosphere environment for rice P uptake (Sun G. et al., 2025).

We further revealed the potential influence of CA on soil biomarkers using a random forest model (Figure 5). The addition of CA significantly enriched *Fusarium* (ASV_67), and *Doassansiopsis* (ASV_24), all of which have multiple pathogenic functions. However, the relative abundances of these fungi were significantly positively correlated with P uptake (Figure 6). This suggests that CA has a positive stimulating effect on both AM fungi and pathogenic fungi, while arbuscular mycorrhizal fungi inhibit the negative harm of pathogenic fungi to rice roots. In detail, AM fungi can develop the mycorrhizal networks to alter the rhizosphere microbiome assembly, facilitating plant disease resistance (Zhang X. et al., 2025). In addition, CA effectively reduced the relative abundance of *Fusarium* (ASV_122) (Figure 5B), one of the main pathogens causing common diseases such as rice blast and bakanae disease (Zhang Q. et al., 2025; Huang et al., 2025). These results indicate that CA has great potential for enhancing disease resistance. In future research, we should attempt to isolate and cultivate *Diversispora* from soil and further validate its ecological functions.

These findings collectively indicate that CA is not only a carbon source but also a powerful microbial community “regulatory switch” (Xie et al., 2019; Feng et al., 2021). The directional regulation of this community structure reflects the dual benefits of CA as a ‘rhizosphere signaling substance'. On the one hand, it enhances P bioavailability by enriching beneficial fungi; on the other hand, inhibiting harmful bacterial communities can effectively reduce the occurrence of rice diseases (Fan et al., 2025; Zhou et al., 2023). CA promotes the synergistic improvement of P uptake efficiency and disease resistance in rice to enhance overall growth.

### 4.3 Strategies and application prospects

Although this study suggests that CA has great potential for regulating microbial communities and promoting P uptake, its application efficiency under field conditions still faces challenges. Firstly, similar to most root exudates, the chemical stability, retention time, and migration range of CA in soil, which directly determine its effectiveness and persistence, remain unclear (Wang and Lambers, 2020; Kretschmer et al., 2022). Therefore, the primary strategy for improving CA efficiency is to optimize its application. For instance, CA could be combined with controlled-release fertilizers, and coating materials can be used to control the release of CA to ensure its sustained effectiveness. Following the fertilizer orientation of crop roots, inducing more effective contact and utilizing P activated by CA in roots can help fully leverage the synergistic effect of “chemical activation” and “physical convergence” (Wen et al., 2019; Liu, 2021). Notably, the bioactivity and P-promoting efficiency of CA may vary drastically under contrasting soil P levels. In moderate-P or low-P soils, CA is expected to exert more pronounced effects on rhizosphere microbial regulation and insoluble P mobilization, as the demand for P activation and beneficial microbial symbiosis is higher in P-deficient environments (Wang et al., 2022). This P level-dependent effect should be fully considered for targeted field application, avoiding inefficient input in high-P farmlands and maximizing CA efficacy in P-limited soils. Secondly, exploring the combined application of CA and specific functional microbial agents (such as AMF agents rich in diverse spores) holds significant promise. This precise regulation strategy of “signal substances-functional microbes” could overcome the limitations of individual approaches, enabling the construction of more stably and efficient beneficial rhizosphere micro ecological systems (Pang and Xu, 2024; Zhuang et al., 2024).

In summary, continuous addition of CA alters soil fungal community structure, increases the relative abundance of *Diversispora* (AMF), and significantly promotes P uptake in rice. While the positive effects of CA were validated in pot experiments, its efficacy may be weakened by factors such as the deep root characteristics of crops, soil heterogeneity, and variable climatic conditions in complex field environments (Wang et al., 2020; Laughlin et al., 2021). Therefore, future research should focus on verifying the effects of CA under different field conditions, soil types, environmental stresses, and gradient soil P levels. Investigating these aspects will not only support the development of CA as a new type of green phosphate fertilizer signaling substance but also improve our understanding of how rhizosphere signaling substances regulate P turnover, a core scientific challenge.

## Data Availability

The bacterial ITS rRNA sequences obtained from this study were deposited in the National Center for Biotechnology In-formation Sequence Reads Archive (SRA accession: PRJNA1450320).

## References

[B1] Backer R. Rokem J. S. Ilangumaran G. Lamont J. Praslickova D. Ricci E. . (2018). Plant growth-promoting rhizobacteria: context, mechanisms of action, and roadmap to commercialization of biostimulants for sustainable agriculture. Front. Plant Sci. 9:1473. doi: 10.3389/fpls.2018.01473 30405652 PMC6206271

[B2] Berendsen R. L. Vismans G. Yu K. Song Y. de Jonge R. Burgman W. P. . (2018). Disease-induced assemblage of a plant-beneficial bacterial consortium. ISME J. 12, 1496–1507. doi: 10.1038/s41396-018-0093-1 29520025 PMC5956071

[B3] Bolyen E. Rideout J. R. Dillon M. R. Bokulich N. A. Abnet C. C. Al-Ghalith G. A. . (2018). QIIME 2: Reproducible, interactive, scalable, and extensible microbiome data science. PeerJ Inc. 6:e27295v2. doi: 10.7287/peerj.preprints.27295v2 PMC7015180 31341288

[B4] Bulgarelli D. Schlaeppi K. Spaepen S. Ver Loren van Themaat E. Schulze-Lefert P. (2013). Structure and functions of the bacterial microbiota of plants. Annu. Rev. Plant Biol.. 64, 807–838. doi: 10.1146/annurev-arplant-050312-120106 23373698

[B5] Callahan B. J. McMurdie P. J. Rosen M. J. Han A. W. Johnson A. J. Holmes S. P. (2016). DADA2: High-resolution sample inference from Illumina amplicon data. Nat. Methods. 13, 581–583. doi: 10.1038/nmeth.3869 27214047 PMC4927377

[B6] Edwards J. A. Santos-Medellín C. M. Liechty Z. S. Nguyen B. Lurie E. Eason S. . (2018). Compositional shifts in root-associated bacterial and archaeal microbiota track the plant life cycle in field-grown rice. PLoS Biol. 16:e2003862. doi: 10.1371/journal.pbio.2003862 29474469 PMC5841827

[B7] Fan X. Ge A. H. Qi S. Guan Y. Wang R. Yu N. . (2025). Root exudates and microbial metabolites: signals and nutrients in plant-microbe interactions. Sci. China Life Sci. 68, 2290–2302. doi: 10.1007/s11427-024-2876-0 40080268

[B8] Feng H. Fu R. Hou X. Lv Y. Zhang N. Liu Y. . (2021). Chemotaxis of beneficial rhizobacteria to root exudates: the first step towards root-microbe rhizosphere interactions. Int. J. Mol. Sci. 22:6655. doi: 10.3390/ijms22136655 34206311 PMC8269324

[B9] He J. Zhang L. Van Dingenen J. Desmet S. Goormachtig S. Calonne-Salmon M. . (2024). Arbuscular mycorrhizal hyphae facilitate rhizobia dispersal and nodulation in legumes. ISME J. 18:wrae185. doi: 10.1093/ismejo/wrae185 39325968 PMC11520417

[B10] Hu L. Robert C. A. M. Cadot S. Zhang X. Ye M. Li B. . (2018). Root exudate metabolites drive plant-soil feedbacks on growth and defense by shaping the rhizosphere microbiota. Nat. Commun. 9:2738. doi: 10.1038/s41467-018-05122-7 30013066 PMC6048113

[B11] Huang Q. Wang R. Ding Q. Liao F. Zhu L. Huang M. . (2025). Low-nitrogen input enriches Massilia bacteria in the phyllosphere to improve blast resistance in rice. New Phytol. 248, 3151–3167. doi: 10.1111/nph.70582 40963442

[B12] Koester M. Stock S. C. Nájera F. Abdallah K. Gorbushina A. Prietzel J. . (2021). From rock eating to vegetarian ecosystems — Disentangling processes of phosphorus acquisition across biomes. Geoderma 388, 114827. doi: 10.1016/j.geoderma.2020.114827

[B13] Kretschmer M. Damoo D. Sun S. Lee C. W. J. Croll D. Brumer H. . (2022). Organic acids and glucose prime late-stage fungal biotrophy in maize. Sci 376, 1187–1191. doi: 10.1126/science.abo2401 35679407

[B14] Laughlin D. C. Mommer L. Sabatini F. M. Bruelheide H. Kuyper T. W. McCormack M. L. . (2021). Root traits explain plant species distributions along climatic gradients yet challenge the nature of ecological trade-offs. Nat. Ecol. Evol. 5, 1123–1134. doi: 10.1038/s41559-021-01471-7 34112996

[B15] Lebeis S. L. Paredes S. H. Lundberg D. S. Breakfield N. Gehring J. McDonald M. . (2015). Salicylic acid modulates colonization of the root microbiome by specific bacterial taxa. Science.. 349:860–863. doi: 10.1126/science.aaa8764 26184915

[B16] Li Y. Lu L. Wang Q. Liu X. Tian J. Zhang R. . (2025). Arbuscular Mycorrhizal Fungi Promote Nodulation and N_2_ Fixation in Soybean by Specific Root Exudates. Plant Cell Environ. 48, 5514–5528. doi: 10.1111/pce.15529 40195807

[B17] Liu D. (2021). Root developmental responses to phosphorus nutrition. J. Integr. Plant Biol. 63, 1065–1090. doi: 10.1111/jipb.13090 33710755

[B18] Liu Y.-W. Guan D.-X. Qiu L.-X. Luo Y. Liu F. Teng H. H. . (2025). Spatial dynamics of phosphorus mobilization by mycorrhiza. Soil Biol. Biochem. 206:109797. doi: 10.1016/j.soilbio.2025.109797

[B19] Martin M. (2011). Cutadapt removes adapter sequences from high-throughput sequencing reads. EMBnet J. 17, 10–12. doi: 10.14806/ej.17.1.200

[B20] Mendes R. Garbeva P. Raaijmakers J. M. (2013). The rhizosphere microbiome: significance of plant beneficial, plant pathogenic, and human pathogenic microorganisms. FEMS Microbiol. Rev. 37, 634–663. doi: 10.1111/1574-6976.12028 23790204

[B21] Olsen S. R. (1954). Estimation of available phosphorus in soils by extraction with sodium bicarbonate. Miscellaneous Paper Institute for Agricultural Research Samaru http://dx.doi.org

[B22] Pang Z. Xu P. (2024). Probiotic model for studying rhizosphere interactions of root exudates and the functional microbiome. ISME J. 18:wrae223. doi: 10.1093/ismejo/wrae223 39495615 PMC11572495

[B23] Sokolova T. A. (2020). Low-Molecular-Weight Organic Acids in Soils: Sources, Composition, Concentrations, and Functions: A Review. Eurasian. Soil Sc. 53, 580–594. doi: 10.1134/S1064229320050154

[B24] Sun G. Li Z. Wang G. Cai H. Yu J. Li Z. . (2025). Infestation by potato tuber moth restructures microbial communities in flue-cured tobacco rhizosphere and non-rhizosphere soils. Front. Plant Sci. 16:1670207. doi: 10.3389/fpls.2025.1670207 41069464 PMC12504494

[B25] Sun N. Wang L. Feng G. (2025). Host-dependent roles of hyphosphere keystone Massilia in organic phosphorus mineralization and AM fungal growth. J. Exp. Bot. eraf339. doi: 10.1093/jxb/eraf339 41030066

[B26] Thomas R. L. Sheard R. W. Moyer J. R. (1967). Comparison of conventional and automated procedures for nitrogen, phosphorus, and potassium analysis of plant material using a single digestion1. Agron. J. 59, 240–243. doi: 10.2134/agronj1967.00021962005900030010x

[B27] Trivedi P. Leach J. E. Tringe S,. G Sa T. Singh B. K. (2020). Plant-microbiome interactions: from community assembly to plant health. Nat. Rev. Microbiol. 18, 607–621. doi: 10.1038/s41579-020-0412-1 32788714

[B28] Wang G. George T. S. Pan Q. Feng G. Zhang L. (2022). Two isolates of *Rhizophagus irregularis* select different strategies for improving plants phosphorus uptake at moderate soil P availability. Geoderma 421:115910. doi: 10.1016/j.geoderma.2022.115910

[B29] Wang G. Yang Y. Liu C. Wang Z. Liu L. Wang X. . (2025a). Simulated plant-derived exudate pulses promote maize phosphorus uptake by recruiting specific rhizosphere microbial communities and shaping root metabolites. Agric,. Ecosyst. Environ. Appl. Soil Ecol.. 213:106244. doi: 10.1016/j.apsoil.2025.106244

[B30] Wang G. Yang Y. Yao Y. Wang X. (2025b). Exudate pulses throughout the entire growth period trigger the increase in maize phosphorus use efficiency by modifying soil keystone microbial taxa. Biol. Fertil. Soils. 61, 999–1011. doi: 10.1007/s00374-025-01912-6

[B31] Wang X. Whalley W. R. Miller A. J. White P. J. Zhang F. Shen J. (2020). Sustainable cropping requires adaptation to a heterogeneous rhizosphere. Trends Plant Sci. 25, 1194–1202. doi: 10.1016/j.tplants.2020.07.006 32830043

[B32] Wang Y. Lambers H. (2020). Root-released organic anions in response to low phosphorus availability: recent progress, challenges and future perspectives. Plant Soil. 447, 135–156. doi: 10.1007/s11104-019-03972-8

[B33] Wang Y. Luo D. Xiong Z. Wang Z. Gao M. (2023). Changes in rhizosphere phosphorus fractions and phosphate-mineralizing microbial populations in acid soil as influenced by organic acid exudation. Soil Till. Res. 225:105543. doi: 10.1016/j.still.2022.105543

[B34] Weber S. E. Bascompte J. Kahmen A. Niklaus P. A. (2025). AMF diversity promotes plant community phosphorus acquisition and reduces carbon costs per unit of phosphorus. New Phytol. 248, 886–896. doi: 10.1111/nph.70161 40248851

[B35] Wen Z. Li H. Shen Q. Tang X. Xiong C. Li H. . (2019). Tradeoffs among root morphology, exudation and mycorrhizal symbioses for phosphorus-acquisition strategies of 16 crop species. New Phytol. 223, 882–895. doi: 10.1111/nph.15833 30932187

[B36] Wilhelm R. C. DeRito C. M. Shapleigh J. P. Madsen E. L. Buckley D. H. (2021). Phenolic acid-degrading Paraburkholderia prime decomposition in forest soil. ISME Commun. 1:4. doi: 10.1038/s43705-021-00009-z 36717596 PMC9723775

[B37] Wu C. Yang Y. Wang Y. Zhang W. Sun H. (2024). Colonization of root endophytic fungus Serendipita indica improves drought tolerance of Pinus taeda seedlings by regulating metabolome and proteome. Front. Microbiol. 15:1294833. doi: 10.3389/fmicb.2024.1294833 38559354 PMC10978793

[B38] Wu J. Liu S. Zhang H. Chen S. Si J. Liu L. . (2025). Flavones enrich rhizosphere Pseudomonas to enhance nitrogen utilization and secondary root growth in Populus. Nat. Commun. 16:1461. doi: 10.1038/s41467-025-56226-w 39920117 PMC11805958

[B39] Xie X. G. Zhang F. M. Yang T. Chen Y. Li X. G. Dai C. C. (2019). Endophytic fungus drives nodulation and n_2_ fixation attributable to specific root exudates. mBio. 10, e00728–e00719. doi: 10.1128/mBio.00728-19 31311876 PMC6635524

[B40] Xie Z. Zhao S. Li Y. Deng Y. Shi Y. Chen X. . (2023). Phenolic acid-induced phase separation and translation inhibition mediate plant interspecific competition. Nat. Plants. 9, 1481–1499. doi: 10.1038/s41477-023-01499-6 37640933

[B41] Zaura E. Keijser B. J. Huse S. M. Crielaard W. (2009). Defining the healthy “core microbiome” of oral microbial communities. BMC Microbiol. 9:259. doi: 10.1186/1471-2180-9-259 20003481 PMC2805672

[B42] Zhang J. Liu Y. X. Zhang N. Hu B. Jin T. Xu H. . (2019). NRT1.1B is associated with root microbiota composition and nitrogen use in field-grown rice. Nat. Biotechnol. 37, 676–684. doi: 10.1038/s41587-019-0104-4 31036930

[B43] Zhang J. Wei Y. Li H. Hu J. Zhao Z. Wu Y. . (2023). Rhizosphere microbiome and phenolic acid exudation of the healthy and diseased American ginseng were modulated by the cropping history. Plants 12:2993. doi: 10.3390/plants12162993 37631203 PMC10459672

[B44] Zhang Q. Liu X. Gu A. Chang X. Shi X. Li X. . (2025). Nano-formulated prothioconazole seed coating improves rice bakanae control and seedling establishment via metabolome and respiration modulation. Chem. Eng. J. 524:169529. doi: 10.1016/j.cej.2025.169529

[B45] Zhang X. Jin X. Li J. Dini-Andreote F. Li H. Khashi U Rahman M. . (2025). Common mycorrhizal networks facilitate plant disease resistance by altering rhizosphere microbiome assembly. Cell Host Microbe. 33, 1765–1778. doi: 10.1016/j.chom.2025.08.016 40961934

[B46] Zheng J. Shi G. Dini-Andreote F. Yang Y. Jiang Y. (2025). Root-derived low molecular weight organic acids modulate keystone microbial taxa impacting plant phosphorus acquisition. J. Adv. Res. S2090-1232(25)00441-2. doi: 10.1016/j.jare.2025.06.032 40533056 PMC12957842

[B47] Zhou X. Zhang J. Khashi U Rahman M. Gao D. Wei Z. Wu F. . (2023). Interspecific plant interaction via root exudates structures the disease suppressiveness of rhizosphere microbiomes. Mol. Plant. 16, 849–864. doi: 10.1016/j.molp.2023.03.009 36935607

[B48] Zhuang Y. Wang H. Tan F. Wu B. Liu L. Qin H. . (2024). Rhizosphere metabolic cross-talk from plant-soil-microbe tapping into agricultural sustainability: current advance and perspectives. Plant Physiol. Biochem. 210:108619. doi: 10.1016/j.plaphy.2024.108619 38604013

