# Supplementary material for: Simulating phenolic acid input enhances rice phosphorus uptake by increasing the relative abundance of Diversispora
Source: Front Microbiol. 2026 Apr 29;17:1810465. doi: 10.3389/fmicb.2026.1810465 (PMC13167988; doi:10.3389/fmicb.2026.1810465)
Supplement: Supplementary file 1 [file Table_1.docx]

**Supporting Information**

Table S1 The keystone ASVs taxonomy of fungi

| ASV | Phylum | Order | Genus |
| --- | --- | --- | --- |
| ASV_67 | Ascomycota | Hypocreales | Fusarium |
| ASV_174 | Ascomycota | Pleosporales | Lophiostoma |
| ASV_24 | Basidiomycota | Urocystidales | Doassansiopsis |
| ASV_206 | Basidiomycota | Agaricales | Coprinellus |
| ASV_192 | Ascomycota | Hypocreales | Trichoderma |
| ASV_63 | Ascomycota | Hypocreales | Varicosporellopsis |
| ASV_117 | Basidiomycota | Auriculariales | Auricularia |
| ASV_122 | Ascomycota | Hypocreales | Fusarium |
| ASV_87 | Ascomycota | Microascales | Scedosporium |
| ASV_52 | Basidiomycota | Cystofilobasidiales | Tausonia |
| ASV_107 | Ascomycota | Hypocreales | Trichoderma |
| ASV_81 | Ascomycota | Candelariales | Pycnora |
| ASV_46 | Ascomycota | Eurotiales | Penicillium |
| ASV_143 | Ascomycota | Saccharomycetales | Suhomyces |
| ASV_41 | Ascomycota | Pleosporales | Paraconiothyrium |
| ASV_125 | Ascomycota | Pleosporales | Phoma |
| ASV_306 | Basidiomycota | Agaricales | Coprinellus |
| ASV_173 | Ascomycota | Pleosporales | Lophiostoma |
| ASV_222 | Ascomycota | Pleosporales | Alternaria |
| ASV_294 | Ascomycota | Microascales | Kernia |
